# Supplementary material for: Strain-gradient mediated local conduction in strained bismuth ferrite films
Source: Nat Commun. 2019 Jun 26;10:2791. doi: 10.1038/s41467-019-10664-5 (PMC6594973; doi:10.1038/s41467-019-10664-5)
Supplement: Supplementary file 1 — Supplementary Information [file 41467_2019_10664_MOESM1_ESM.pdf]

Supplementary Information

for

Strain-Gradient Mediated Local Conduction in Strained Bismuth Ferrite Films

Ming-Min Yang et al.

Supplementary Note 1: Conduction process of the conductive AFM measurement

The morphotropic and ′-phases in the strained $BiFeO_3$ thin films combine epitaxially with each other in an alternating way with a tilting angle (see Supplementary Figure 1). As both morphotropic phases possess certain conduction, they will always get involved in the current conduction to certain extend regardless of whether the AFM tip contacts -phase or ′-phase. Specifically, when the AFM tip contacts the middle of the less conductive -phase, the tip probes a low current (Supplementary Figure 1a); when the tip moves towards the ′-phase, current would also flow in the conductive ′-phase after passing the less conductive -phase, leading to an increased current magnitude (Supplementary Figure 1b); When the tip contact directly at the middle of ′-phase, the dark current reach to its maximum value, thus forming the previously mentioned saw-like features. The plateaus in the scan profile would appear if one of the phases in completely insulating, thus blocking current injection from the AFM tip when the tip only contacts this phase. This occurs for example in the AFM scanning on the conductive domain walls embedded in the insulating domain matrix. [1] Furthermore, even in the case that the phase boundaries is more conductive than the morphotropic phases, the cAFM scan profile would also show the saw-like feature but with maxima appearing directly at the boundary locations. Clearly, this is not the case in our work. Thus, it is justified to conclude that the morphotropic ′-phase possesses an enhanced dark conduction compared to that of the -phase.

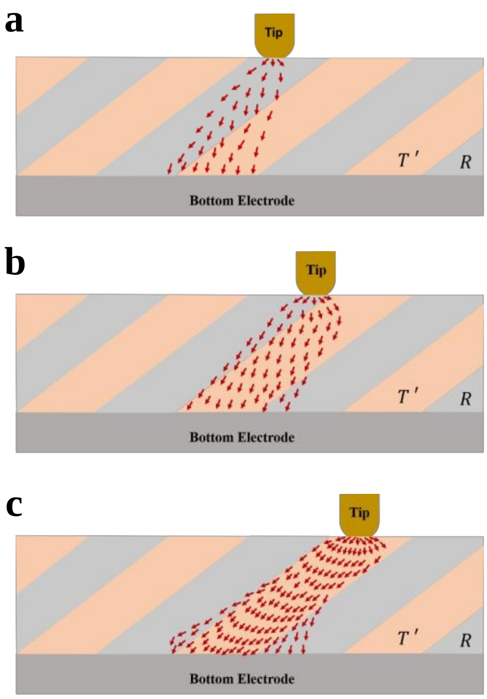

Supplementary Figure 1| Schematics shows the current flow path under the conductive AFM tip. (**a**) The conductive AFM tip contacts the -phase, (**b**) the boundary region and (**c**) ′-phase measured in the dark condition. AFM denotes the atomic force microscopy.

Supplementary Note 2: Spatially resolved dark current distribution on strained $BiFeO_3$ film

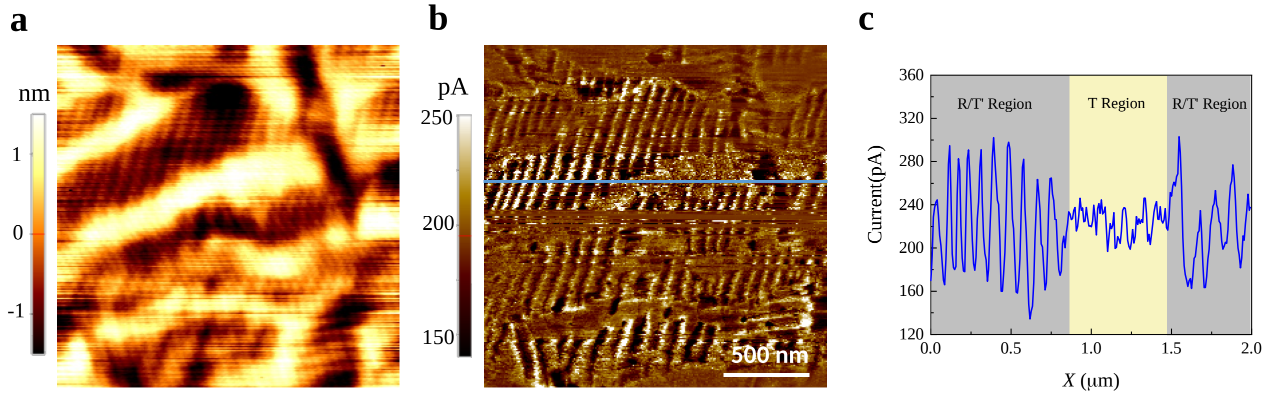

Supplementary Figure 2| Spatially resolved dark current distribution. (**a**)Surface topography and (**b**) corresponding dark current distribution. (**c**) Dark current and surface morphology profile comparison of the area marked by blue line in (b). The measurement condition used here is the same as that in Figure 1 of the main text.

Supplementary Note 3: *I-V* characteristics measured under illumination by Ph-AFM

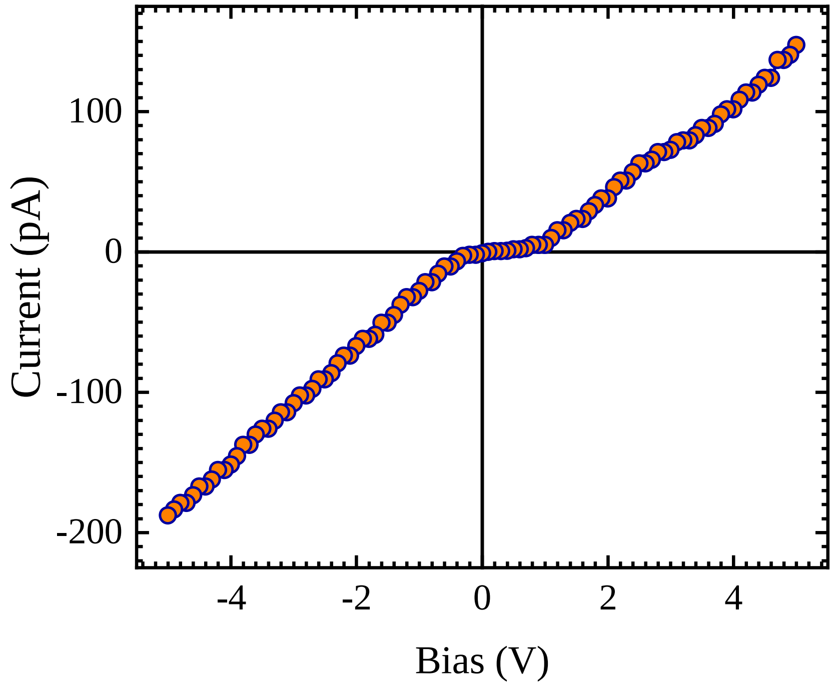

Supplementary Figure 3| Photo I-V curve probed by the Ph-AFM tip under illumination of 405 nm light. The light intensity is 1 W • cm^{-2} .

Supplementary Note 4: Photocurrent mapping with or without external bias

Although the strained $BiFeO_3$ film possesses a potential photovoltaic effect, the conductive AFM tip probes negligible current under illumination without applying external bias, as demonstrated in Supplementary Figure 4. The photocurrent appears only if an external bias is applied. Thus, the current and its variation detected by the conductive tip under illumination are solely due to local photoconduction driven by external bias rather than photovoltaic or any combination effects.

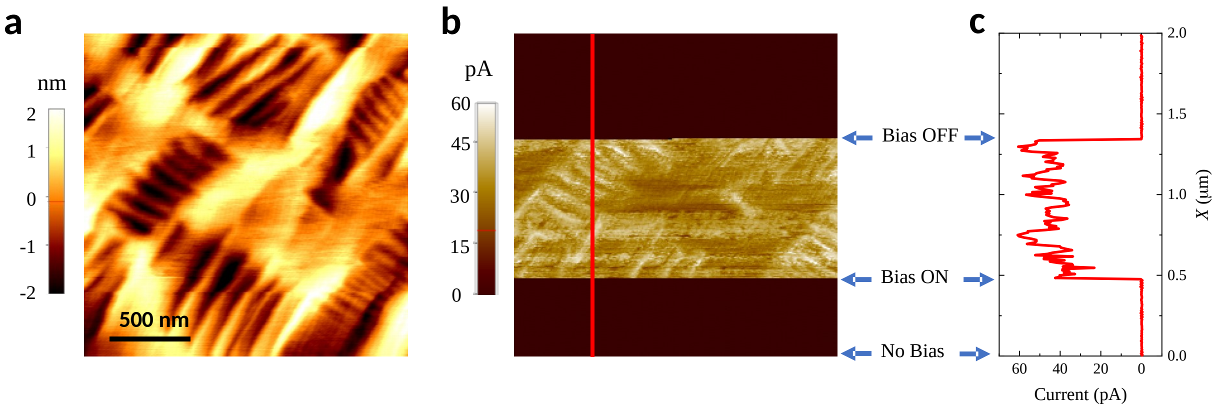

Supplementary Figure 4| Role of external bias in the mapped photocurrent. (**a**) Scanned topography and (**b**) the corresponding current mapping. The time when external 2V bias is turned on or off is shown nearby the image. (**c**) Current profile on the scan area marked by red line in (b).

Supplementary Note 5. The role of the optical absorption variation in the observed photo-conduction contrast

Due to their different crystallographic structures, the morphotropic and ′ phases are likely to possess different bandgap and light absorption coefficients. Thus, it is important to understand the possible role of the light absorption variation in mediating local photoconductions.

The lattice structure of the phase is relaxed in comparison to that of the ′ phase, resulting in lattice parameters similar to that of $BiFeO_3$ films grown on less strained substrate, such as LAST. It would be reasonable to assume that the phase possess similar bandgap and optical absorption coefficient to that of rhombohedral $BiFeO_3$ film ($= 2.5 \times 10^{5}\ \mathrm{cm}^{-1}$ for 405 nm light).[2] The light absorption coefficient of the matrix -phase and the morphotropic ′-phase under the illumination of 405 nm laser equals to $= 1.23 \times 10^{5}\ \mathrm{cm}^{-1}$. Given the film thickness as 100 nm, the -phase would absorb 92% of the light with 8% of the light transmitted. In the case of the matrix -phase and the morphotropic ′-phase, about 70% of the light is absorbed with 30% light transmitted. Note that the light reflection by the film surface is assumed to be homogenous over different phases and is not considered here. Thus, the -phase absorbs 1.3 times of light than that of matrix -phase and the morphotropic ′-phase under the 405 nm laser illumination. Provided that the density of photo-excited carriers is proportional to the absorbed light intensity, more non-equilibrium carriers are excited in the -phase than those in the matrix - and the morphotropic ′-phase. Note that this light-absorption variation itself would not induced space charge field as each part of the phase is still in neutral charge (see Supplementary Figure 5a). Due to the higher density of non-equilibrium carriers in the -phases, non-equilibrium carriers diffuse from the -phase to the ′-phases, resulting in space charge of which field prevents further carrier diffusion (see Supplementary Figure 5b). Consequently, the density of non-equilibrium carriers in the ′-phase is increased, resulting in

$$_{\mathrm{R-phase}} > \ _{\mathrm{T'-phase}} > \ _{\mathrm{T-phase}} \qquad 1$$

where is the density of non-equilibrium carriers. As the conduction is proportional to the density of carriers, this would give rise to the photoconduction contrast as

$$_{\mathrm{R-phase}} > \ _{\mathrm{T'-phase}} > \ _{\mathrm{T-phase}} \qquad 2$$

Clearly, this is inconsistent with experimental observation presented in the manuscript wherein $_{\mathrm{R-phase}} > \ _{\mathrm{T-phase}} > \ _{\mathrm{T'-phase}}$. Thus, the light absorption contrast between morphotropic phases cannot account for the photoconduction contrast experimentally observed.

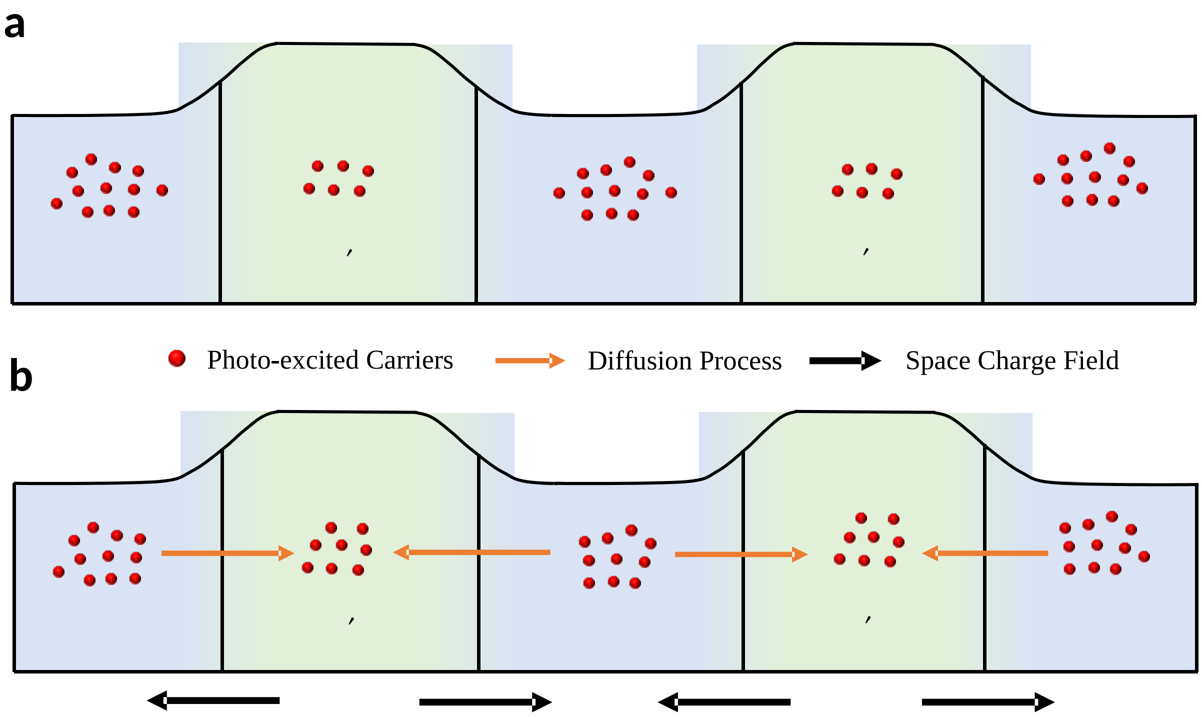

Supplementary Figure 5| Inhomogeneous light absorption induced diffusion process. (**a**) Schematic shows the variation of the light-induced nonequilibrium carrier density in the morphotropic phase region due to the stronger light absorption in the -phase under 405 nm laser illumination. Here, the diffusion process is not yet considered. (**b**) Diffusion induced non-equilibrium carrier redistribution and associated space charge field. Here, the photo-excited carriers are assumed to be holes, resulting in a space charge field pointing from ′-phase to the -phase.

To further demonstrate the crucial role of the flexo-photovoltaic effect and especially, to exclude the effect of light absorption variation, we mapped spatial distribution of the photoconduction over $BiFeO_3/LaAlO_3$ thin film under the illumination of 365 nm light. According to Chen et al., the less-strained rhombohedral $BiFeO_3$ and the strained $BiFeO_3/LaAlO_3$ thin films exhibit similar absorption coefficient of 365 nm light ($\approx 2.8 \times 10^{5}$ cm^{-1}).[2] Thus, it would be reasonable to assume a homogenous light absorption over the morphotropic phases in the strained $BiFeO_3$ films. However, as shown in Supplementary Figure 6, there exists clear photocurrent contrast over the scanned area consisting of matrix -phase, morphotropic ′-phase and -phase. As profiled in Supplementary Figure 6c, the current probed at the -phase is about three times larger than that collected at the nearby ′-phase. Therefore, the light absorption variation in the strained $BiFeO_3$ thin film do not play a major role in tuning local photoconduction.

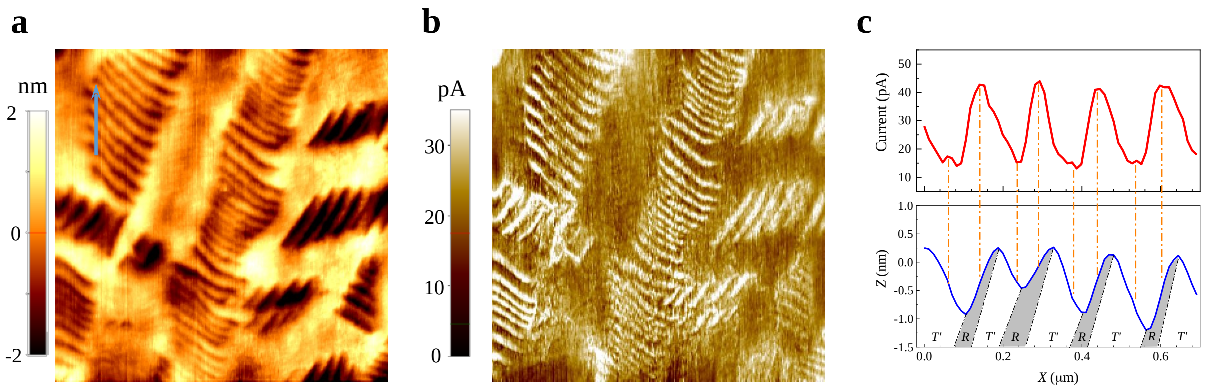

Supplementary Figure 6| Spatially resolved photocurrent distribution under 365 nm light illumination. (**a**) surface topography and (**b**) Photocurrent distribution characterized under illumination of 365 nm light; (**c**) Profile comparison between the photocurrent and surface morphology of the area marked by blue arrow in (a). The light intensity is about 200 mW/cm^2 and a bias of 3 V is applied to the side Pt electrode.

Owing to the tilting of the ferroelectric polarization away from the surface normal direction (i.e. $[001]_{pc}$), both morphotropic phases (namely, -phase and ′-phase) in principle possess certain light polarization dependent absorption. Although we have confirmed above that the light absorption difference between morphotropic phases do not account for the observed photoconduction contrast, the potential impact of its light-polarization dependence on local photoelectric properties deserves further exploration. To this end, we studied the light polarization dependent photoconduction of $BiFeO_3/LaAlO_3$ (001) thin film with illumination along the surface normal direction. Here, the $BiFeO_3/LaAlO_3$ (001) film consists solely of monoclinic -phase with a thickness of 40 nm and the in-plane electrodes are perpendicular to the in-plane ferroelectric polarization (see Supplementary Figure 7a). As shown in Supplementary Figure 7b, the in-plane photoconduction of the -phase $BiFeO_3$ stays almost constant while rotating the incident light polarization, probably due to the small mismatch angle between ferroelectric polarization and surface normal direction in $BiFeO_3/LaAlO_3$ films. Assuming no dependence of carrier mobility on the light polarization, the above result points to negligible light polarization dependence of the photo-excited carrier density in -phase $BiFeO_3$. This is also consistent with previous report using out-of-plane capacitor geometry (see ref. 5).

In the case of morphotropic -phase in the mixed region, it is difficult to directly probe the intrinsic light polarization dependence of its photoelectric properties due to its small dimension and strong interaction with its surroundings. To circumvent this difficulty, we studied the less strained rhombohedral $BiFeO_3/SrTiO_3$ (001) film consisting of a mono-domain structure. Details about this sample can refer to our previous work.[3] Supplementary Figure 7c illustrates the measurement

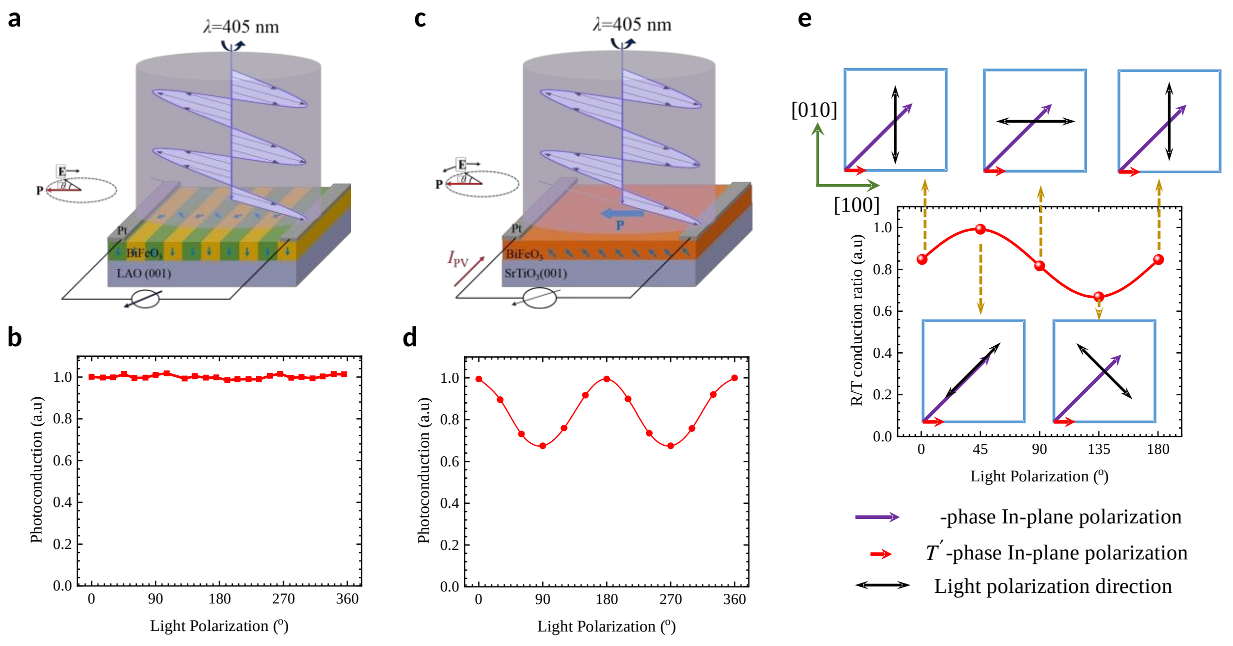

Supplementary Figure 7| Effect of light polarization dependent optical absorption on the $BiFeO_3$ with different structures. (**a**) Schematic shows the measurement geometry and (**b**) the corresponding light polarization dependence of the photoconduction in $BiFeO_3/LaAlO_3$ thin films consisting of solely -phase. (**c**) Schematic shows the measurement geometry and (**d**) the corresponding light polarization dependence of the photoconduction in $BiFeO_3/SrTiO_3$ (001) thin films consisting of a single ferroelectric domain. In (a)-(d), the light polarization angle refers to that made between light polarization and the in-plane net polarization direction. (**e**) Schematic illustrates the light polarization dependence of the photoconduction ratio between and -phases if it is the light polarization dependence of the optical absorption that determines local photoelectric properties while tailoring the light polarization angle.

configuration wherein the in-plane electrodes run perpendicular to the in-plane ferroelectric polarization and light incidents along the surface normal direction. As shown in Supplementary Figure 7d, the photoconduction of the $BiFeO_3/SrTiO_3$ (001) film exhibits strong light polarization dependence. The photoconduction reaches it maxima while light polarization running parallel to the in-plane ferroelectric polarization; whereas, it reduces to its minima when light polarization is perpendicular to the ferroelectric polarization. Note that the ferroelectric polarization of morphotropic -phase in the strained $BiFeO_3/LaAlO_3$ films also tilts towards the $[111]_{pc}$ direction as in the case of the rhombohedral $BiFeO_3/SrTiO_3$ films. It is likely that the -phase shows the similar dependence of photoelectric properties on light polarization with probably a smaller amplitude in variation. Thus, -phase would possess the maximum photoconduction when light polarization runs along its in-plane ferroelectric polarization.

If it is the light polarization dependence of optical absorption that determines the local photoconduction variation while rotating the light polarization (see Figure 4 of the Manuscript), the photoconduction contrast between the -phase and the -phase should maximize when light polarization is parallel to the in-plane ferroelectric polarization of -phase (i.e. = 45 °). Afterwards, it reaches a minimum value while light polarization equals to = 135 °, as schematically illustrated in Supplementary Figure 7e. Apparently, this is inconsistent with experimental result shown in Figure 4 of the Manuscript. Therefore, we can conclude that the polarization dependent optical absorption also do not play a major role in the mediating local photoconduction. Instead, as demonstrated in the Manuscript, it is the flexo-photovoltaic manifested at the morphotropic phase boundaries that controls local photoelectric properties in the mixed phase region.

Supplementary Note 6: Domain structure of the morphotropic region

Owing to the ferroelectric nature of the morphotropic phases in the strained $BiFeO_3$ thin film, both *R*-phase and ′-phase possess non-centrosymmetry and exhibit the bulk photovoltaic effect, which is also able to separate light-excited electron-hole pairs. The domain structure of a morphotropic phase region is characterized by piezoresponse force microscopy as shown in Supplementary Figure 8a-c. In the out-of-plane direction, the strained $BiFeO_3$ film shows a uniform polarization direction pointing towards the substrate. In the in-plane direction, the polarization is aligned toward the right side of the scanned area. As schematically shown in Supplementary Figure 8d, the “unidirectional polarization feature” refers to that each -phase matrix in a morphotropic phase region possess the same polarization distribution and the ′-phase matrix also exhibits the same polarization features but different from that of -phase, which is consistent with previous reports.[4,5] In contrast to the simple domain patterns in the and ′ phase, domain configuration in the morphotropic phase boundaries

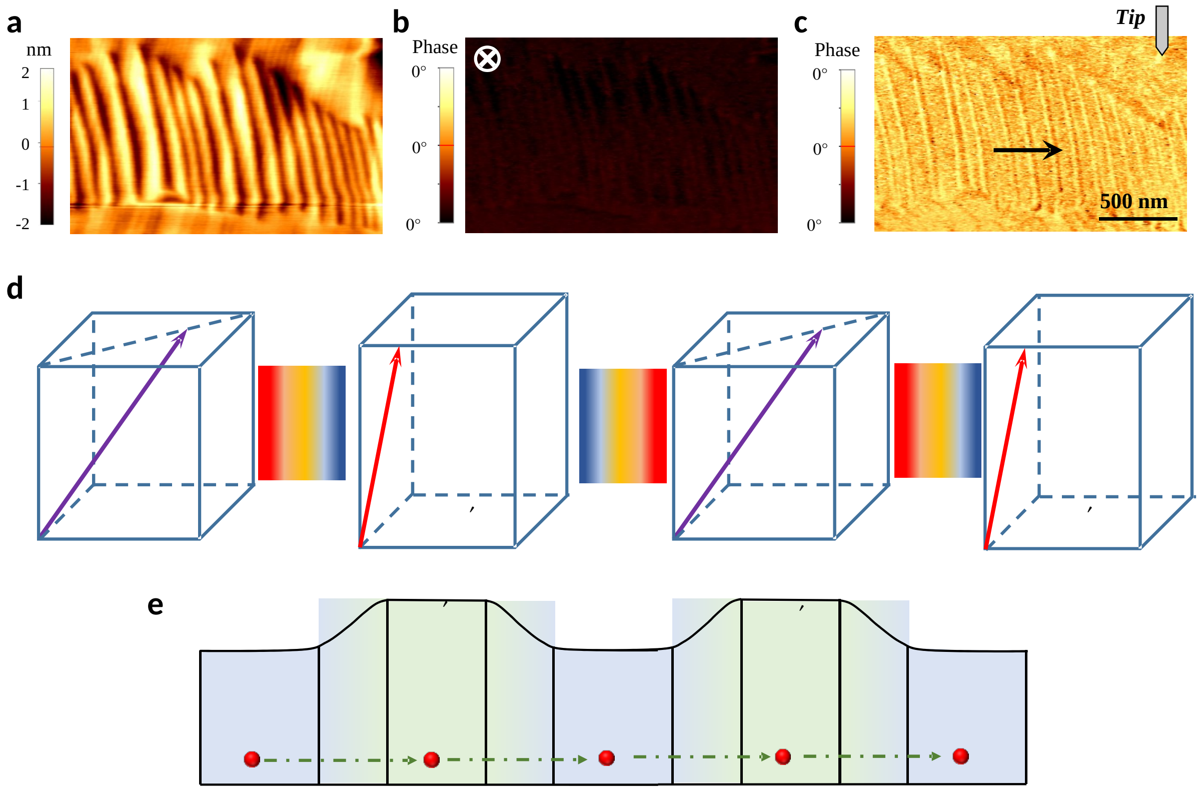

Supplementary Figure 8| Domain structure of the morphotropic region in strained $BiFeO_3$ thin film. (**a**) Topography, (**b**) out-of-plane PFM phase, (**c**) in-plane PFM phase, (**d**) schematic of the ferroelectric polarization in the morphotropic ′ and phases. PB denotes the morphotropic phase boundary. (**e**) Schematic showing the intrinsic bulk photovoltaic effect in the morphotropic phase region. Red ball denotes the carriers delivered by the bulk photovoltaic effect of R-phase.

is very complicated probably due to its complicated structures connecting two types of morphotropic phases and strain gradient induced flexoelectric effect. Also, detailed characterization of the domain structures in the phase boundaries is challenging due to its small dimension and vulnerable structure which is very sensitive to external bias.[6]

Overall, without taking the strain gradient-induced flexo-photovoltaic effect manifested at the phase boundaries into consideration, the intrinsic bulk photovoltaic effect manifested in the *R*-phase would not modulate the non-equilibrium carrier density and photoconduction of nearby ′-phase, as schematically shown in Supplementary Figure 8e. Likewise, the bulk photovoltaic effect of the ′-phase would not alter the photoconduction of nearby -phase.

Supplementary Note 7: Possible role of the depolarization field in mediating local conduction

The strain gradient not only induces the flexo-photovoltaic effect but also the flexoelectric polarization in the morphotropic phase boundary. The flexoelectric polarization would induce a depolarization field only if the polarization is not fully compensated and polarization divergence -∇ • appears. In this case, the photo-excited non-equilibrium carriers at the morphotropic phase boundary would not only subject to the flexo-photovoltaic effect but also the depolarization field. However, the appearance of the depolarization field at morphotropic phase boundary is still speculative. The spatial distribution of the ferroelectric polarization and the corresponding polarization divergence in the morphotropic phase region had been mapped in ref. 5 based on the assumption that electrical polarization is linearly proportional to the piezoresponse vector. This assumption is, however, not applicable to the morphotropic phase boundaries. The large piezoresponse therein is due to the subtle and intricate transformation between those morphotropic phases each with its own symmetry and piezoelectric tensor, rather than a simple proportionality on the magnitude of the ferroelectric polarization.[6] At a more detailed analysis, the derived distribution of the polarization divergence ∇• and the associated depolarization field in the morphotropic phase region is inconsistent with the symmetry of the strain gradient distribution. As shown in Figure 3 of our manuscript, the strain gradient shows a mirror-type symmetrical distribution in morphotropic phase region. Specifically, strain gradient $_{yy}/ l$ at the boundaries located at the right and left side of the ′-phase is always pointing to the ′-phase (see Supplementary Figure 9a). Thus, the resultant flexoelectric polarization and associated depolarization field, if any, would also exhibit similar mirror like symmetry, namely pointing to the opposite direction with respect to the ′-phase (see Supplementary Figure 9b,c; also see ref. 7). By the contrary, the depolarization field at the nearby phase boundaries derived in the ref. 5 is always to be in the same direction (see Supplementary Figure 9d).

Moreover, if the depolarization field exists at the morphotropic phase boundaries due to the large polarization divergence ∇• , this polarization divergence would also induce charge accumulation at the phase boundaries, leading to an enhanced conduction at the boundaries, as in the case of ferroelectric domain walls.[8,9] However, the dark cAFM characterization shown in Figure 1 of the main manuscript did not show any conduction enhancement at phase boundaries. Therefore, it would be reasonable to claim that the polarization divergence and depolarization field is minimal at the morphotropic phase boundary. Thus, the existence and the manifestation of the depolarization field

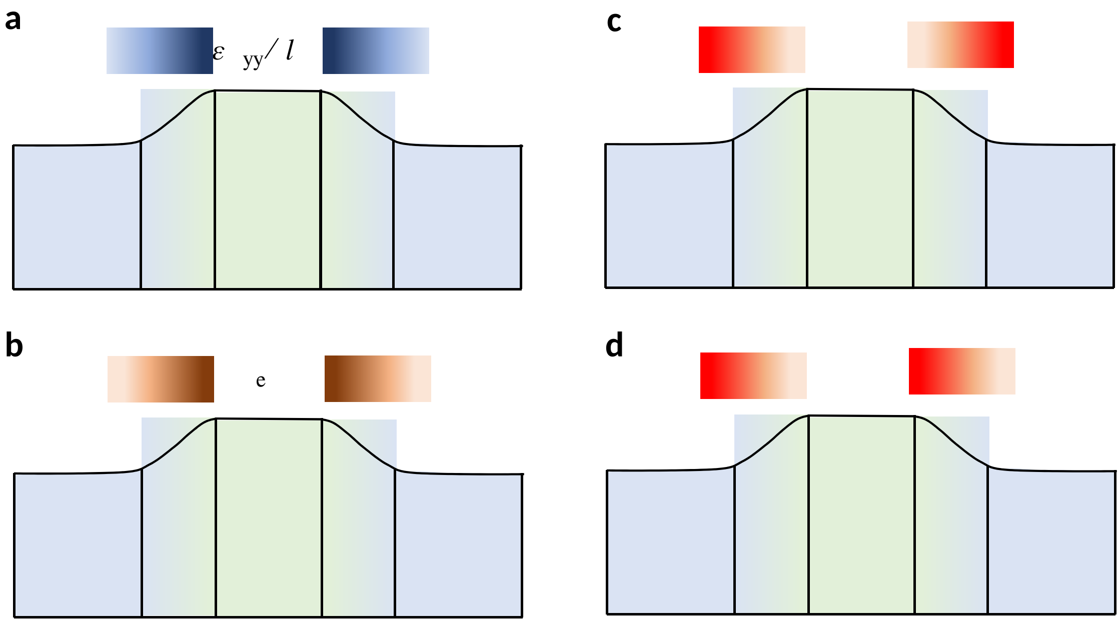

Supplementary Figure 9| Symmetry of the strain gradient distribution and associated effects. Directions of (a) strain gradient, (b) polarization induced by flexoelectric effect and (c) associated depolarization field at nearby morphotropic phase boundaries enclosing the ′-phase. (d) The depolarization field directions derived in the ref. 5.

at the morphotropic phase boundaries is highly questionable and speculative and do not play a major role in mediating local photoelectric properties.

Supplementary Note 8: The photo-conductance ratio between the morphotropic phases and matrix *T*-phase

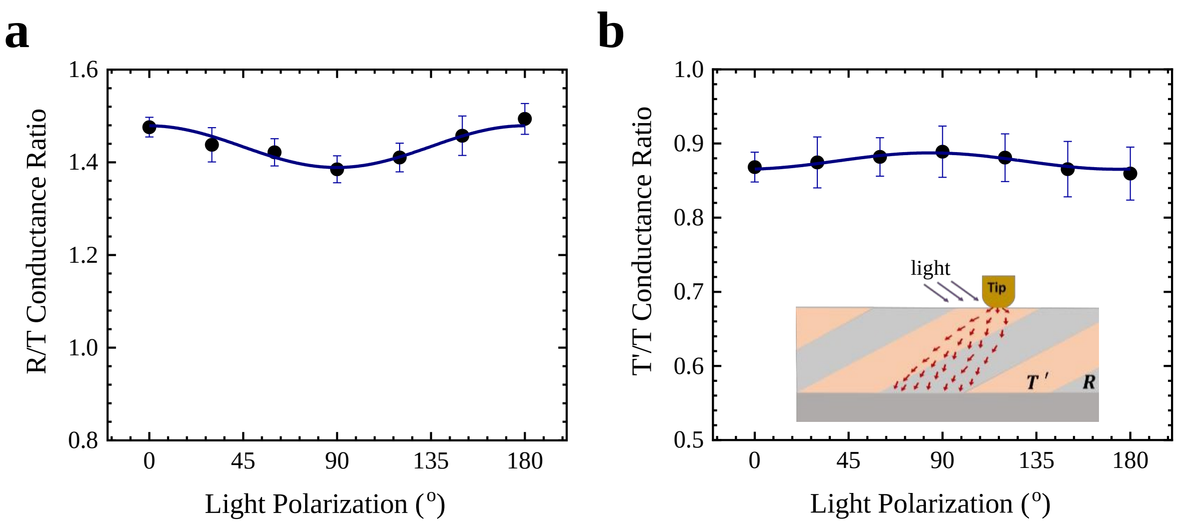

Supplementary Figure 10| Light polarization dependent local photoconduction. (**a**) The photo-conductance ratio between -phase and -phase; (**b**) ′-phase and -phase. The solid curves are the fits of the experimental data with Eq. 1. The error bars indicate the standard deviations of photo-conductance ratio between morphotropic phases mapped in a 2×0.4 μm ^{2} area.

As shown in Supplementary Figure 10a, the photoconduction ratio between morphotropic -phase and the matrix -phase shows sinusoidal dependence on the light polarization, which can be well fitted by Eq. 1 of the manuscript. The photoconduction ratio between ′ and matrix -phase also depends on the light polarization but with a 0° phase shift compared to that of ⁄ conductance ratio. This is also consistent with our proposed model. Note that the variation amplitude of the ′⁄ photocurrent ratio is much smaller than that of the ⁄ ratio. This is probably due to the entangled conduction path underneath the AFM tip. As illustrated in the inset of Supplementary Figure 10b, the nearby -phase with enhanced conductance would also contribute to the conduction when the tip contacts the ′-phase with depressed photoconduction. The enhanced photoconduction of the -phase would compensate somehow the reduced photoconduction of the ′-phase while rotating the light polarization, resulting in a small variation amplitude. Due to the small variation in the amplitude of the ′⁄ conductance ratio, the light polarization dependent conductance ratio between ⁄ ′ shown in the Figure 4 of the manuscript can also be well fitted with Eq.1.

References

1. Volk, T. R., Gainutdinov, R. V., Zhang, H. H. Domain-wall conduction in AFM-written domain patterns in ion-sliced $LiNbO_3$ films, *Appl. Phys. Lett.* **110**, 132905 (2017);

2 Chen, P. *et al*. Optical properties of quasi-tetragonal $BiFeO_3$ thin films *Appl. Phys. Lett*. **96**, 131907 (2010);

3 Yang, M.M., Luo, Z.D., Kim, D.J., Alexe, M. Bulk photovoltaic effect in monodomain $BiFeO_3$ thin films. *Appl. Phys. Lett*. **110**, 183902 (2017);

4 Chen, Y.-C. *et al*. Electrical Control of Multiferroic Orderings in Mixed-Phase $BiFeO_3$ Films *Adv. Mater*.**24**, 3070-3075 (2012);

5 Chu, K. *et al.* Enhancement of the anisotropic photocurrent in ferroelectric oxides by strain gradients. *Nat. Nanotech*. **10**, 972-979 (2015);

6 Zhang, J. X. *et al.* Large field-induced strains in a lead-free piezoelectric material. *Nat. Nanotech*. **6**, 98-102 (2011);

7 Li Y.-J. *et al.*, Mechanical Switching of Nanoscale Multiferroic Phase Boundaries. *Adv. Funct. Mater*. **25**, 3405-3413 (2015);

8 Seidel, J. *et al.*, Conduction at domain walls in oxide multiferroics. *Nat. Mater*. **8**, 229-234 (2009);

9 Crassous, A., Sluka, T., Tagantsev, A.K., & Setter, N. Polarization charge as a reconfigurable quasi-dopant in ferroelectric thin films. *Nat. Nanotech.* **10**, 614-618 (2015);
